# Outpost Hospitals in Large Towns

**Published:** 1893-03-18

**Authors:** 


### The Institutional Workshop.

### HOSPITAL ADMINISTRATION.

### OUTPOST HOSPITALS IN LARGE TOWNS.

(By Our Special Commissioner.)

It will be within the remembrance of many readers that in the course of the evidence given before the Lords' Committee Mr. Burdett laid great stress upon the importance of establishing outpost hospitals or receiving wards in connection with the principal general hospitals in all large towns, and especially in London. Instances were adduced of the injury which patients experience by being brought long distances to the hospitals when suffering from accidents. It was further urged that the expense to the poor connected with the present system, or want of system, whereby three-fourths of the hospital beds available for the poor in the metropolis are located within a two-mile radius of Charing Cross, imperatively needed alteration. Apart from the expense, the suffering and inconvenience, the loss of time, and the difficulties of transit at present weigh heavily on the suffering poor in London, and so ought to secure new arrangements, conceived with a far greater regard to the dictates of humanity, than those now available. The sites of the Poor Law Infirmaries, as well as those of the Fever and Infectious Hospitals under the Metropolitan Asylums Board, are all placed in convenient situations, within easy access of the more crowded districts where the poor reside. It must evidently be a wrong principle that the voluntary hospitals, which are, after all, nothing if they are not charities, should be so situated as to compel the majority of the patients who resort to them to incur loss and hardship because the sites on which they stand are situated miles and miles away from the homes of the majority of the poor. In North London a successful attempt has been made to rectify the error committed by the founders of the older hospitals by moving the Great Northern Hospital, formerly situated in the Caledonian Road, to Islington, so that it is within easy access of the vast majority of the people resident in the district which it serves. Again, in South-East London, where there was formerly no hospital between Woolwich and London Bridge, several small hospitals have recently been established, the Miller Memorial Hospital at Greenwich and the St. John's Hospital at Lewisham being the most important of the number. We are strongly of opinion that the larger hospitals ought to follow the example of the Dreadnought Seamen's Hospital by planting outpost hospitals or receiving houses on convenient sites in the centre of the denser portions of the district which each serves. The branch or outpost hospital connected with the Dreadnought at the East and West India Docks, and the dispensaries at Wells Street and Gravesend, have done immense service to sick seamen, and the success attending these efforts should attract and fix the attention of the managers of every well-conducted general hospital in the metropolis. If some steps are not taken in relation to outpost hospitals in London, and in some of the larger cities of England, the time will come when public opinion will demand the removal of some of the older hospitals from their present sites to others more conveniently situated so far as the patients are concerned. We look forward to the time when we shall have in London a sufficient number of outpost hospitals for the reception of severe accident cases, working under the control of the larger general hospitals, where they can be at once attended to, and from which they can be brought in due course, if thought desirable, on a proper ambulance to the parent institution.

#### An Objection Considered.

The only objection which has been urged to this proposal is that it might unduly interfere with the work of the medical school. A close examination of the experience gained at the branch hospital in connection with the Dreadnought, and a due consideration of this aspect of the question in all its bearings, leads us to conclude that in practise there is nothing material in a criticism of this kind. The medical schools would pursue their work as they do now, unless or until a master-mind arises who will give his giant's strength to the development and carrying out of a scheme for one great medical school or university for the whole of London. Until this could be accomplished, the outpost hospital would materially aid the teachers in the medical schools, because they would ensure that many important cases, which now do not always come to the hospitals, would be received at the outpost institutions, and in due course find their way into the clinical wards.

#### The Condition of Boston, U.S.A.

In this connection, the report of the trustees of the Boston City Hospital on the advisability of establishing cottage or branch hospitals in the several wards of the city, will be read with special interest. It appears that so long ago as 1891 the City Council instructed the Board of Trustees to consider the advisability of establishing branches of the City Hospital in three districts of the city, as part of a comprehensive plan to provide for its future growth. Subsequently similar instructions were given in other districts, and estimates were asked for, showing the cost of building and maintaining such hospitals. The trustees made a careful investigation into the condition and situation of the various hospitals located in Boston, their situation relative to the different portions of the city and the needs which they supply. Inquiries were extended to an examination of the policy pursued by other cities in America and Europe, and to the relative cost of small and large hospitals. In submitting their report, after two years' careful investigation, the trustees considered the question under three aspects, for each district: (1) The small but complete General Hospital; or (2) the Emergency Hospital, where patients should be temporarily received and treated, and when necessary sent forward to the parent hospital; (3) the Hospital Station, for imparting first aid to the sick and injured, and for assisting them to reach the central hospital.

#### Complete Local Hospitals.

This term is applied to a small but complete hospital, adequate for the sick and injured of the district in which it is located, but subject in its general management to the Central Hospital Committee.

The buildings should be substantially erected, in accordance with the best modern requirements for hospital construction, and should be sufficiently large to meet, for a reasonable period to come, the probable increased needs of the community for which it is designed. After exhaustively setting out the present hospital accommodation existing in the City of Boston, to which we hope to return on a future occasion, showing by statistics of accident cases at the two largest hospitals the localities where the injuries were received, and comparing the results with the hospital accommodation afforded by other cities, the trustees deal with the cost of such establishments. This they estimate for five local hospitals, including the purchase of land, buildings, furniture, and equipments, would amount to 700,000 dols. (£140,000), or £28,000 for each hospital. The trustees then bring out the advantages derived by patients who have the benefit of the varied resources which a great hospital can bring to bear upon the cure of disease. "The greater the hospital, if properly managed, the greater must be the facilities afforded. In keeping pace with the times it requires all the improved appliances and methods as they appear. It commands for its service the leaders in every department, in medicine and surgery, and for difficult cases receives the attention of the best talent which the city, and indeed the country, can afford. It attracts, on account of its greater opportunities for observation and study, the best house physicians for the immediate attendance upon the patients, and for their care it can choose the best nursing assistance. So long, therefore, as it is possible to afford reasonably convenient access to a great central hospital, its superior facilities of treatment and cure should, in the opinion of the trustees, be extended alike to the citizens of every section of the city needing its care. The trustees are therefore of opinion that any local benefits which might arise from the establishment of local hospitals in outlying districts, would not be sufficient to offset their disadvantages. They therefore express the opinion that no necessity exists for the establishment of local hospitals, and they regard such a step as unadvisable."

Emergency (Outpost) Hospitals.

Emergency hospitals are temporary hospitals, with from ten to twelve beds each, which admit emergency cases, probably accidents, for temporary treatment and for transference, in the more serious cases, to the large parent hospital for permanent treatment. There is only one city, New York, which at present maintains at the public expense emergency hospitals of this class. There are three such hospitals in New York City, but none of them are intended for the continued treatment of disease or injury, though patients are sometimes retained for a considerable period. Mention is also made of one or two private institutions, and those connected with the Dreadnought Seamen's Hospital, Greenwich. The medical men consulted were opposed to this class of emergency hospitals for various reasons, which I have given at length. The manager under whose care the emergency hospitals of New York are placed, declares the management to be attended with great difficulty and annoyance, owing to the tendency of the local stations to use the emergency hospitals as schools of medical observation, and to retain, through favouritism or for other reasons, cases which should be transferred to the central hospital. The trustees were decidedly of opinion that emergency hospitals of the class considered are not advisable.

Hospital Stations and Ambulances.

The trustees appreciate the fact that the inhabitants of the outlying districts of Boston are at considerable disadvantage as to hospital accommodation as compared with those in the central portions of the city, and make the following proposals: "Being of opinion that the present ambulance service is inadequate to the necessities of the citizens, they advise the extension of the present system to the outlying districts, and recommend the establishment of a hospital station in each district, which shall be in communication with the ambulance, and to which accidents or emergency cases may be taken for immediate treatment, with a view to permanent provision. Each station is to be provided with two or three beds, all suitable appliances, and a trained nurse to care for, and afford to the sufferer the necessary first aid. Daily, at a fixed hour, one of the admittance staff of the Boston City Hospital will attend to investigate applications for admission to the City Hospital. The local relief staff will consist of two medical men to be attached to each station, with which their residences shall be connected by telephone. The trustees further report that experience may warrant the appointment of a skilled house officer from the Boston City Hospital to be detailed for service at each station, with the possible provision for the treatment of out-patients there also, whilst the ambulance work is to be done by members of the Police Department. The charge and care of the station hospitals is to be entrusted to the Boston City Hospital department. They suggest that one such station hospital should be opened immediately, in order to ascertain experimentally the best methods for their establishment and management before proceeding with further stations. With this object they suggest, failing suitable quarters being found in some existing building, that a small house of eight or ten rooms should be taken, which might be furnished and fitted for about £600. The trustees estimate that such a station might be fully maintained at about a cost of £1,000 per annum, and they recommend a vote of 10,000 dols. (£2,000), to cover the expense of the establishment and maintenance of one station for a year. Finally the trustees express the belief that "unnecessarily to divide hospitals is retrogression, and that to strengthen and concentrate hospital treatment is progress in what most vitally concerns the well-being of every citizen."

This report is most able and suggestive. It deals with questions which must, sooner or later, become of pressing importance to the inhabitants of all great cities, and we feel that on the whole the conclusions arrived at are sound, and that they deserve, as they will no doubt receive, the close attention of municipal authorities and of managers of hospitals all the world over.

